# A Ferroptosis-Related Gene Prognostic Index Associated With Biochemical Recurrence and Radiation Resistance for Patients With Prostate Cancer Undergoing Radical Radiotherapy

**DOI:** 10.3389/fcell.2022.803766

**Published:** 2022-02-10

**Authors:** Dechao Feng, Xu Shi, Qiao Xiong, Facai Zhang, Dengxiong Li, Wuran Wei, Lu Yang

**Affiliations:** Department of Urology, Institute of Urology, West China Hospital, Sichuan University, Chengdu, China

**Keywords:** ferroptosis-related gene prognostic index, prostate cancer, tumor immune microenvironment, biochemical recurrence, immune checkpoint

## Abstract

**Background:** Ferroptosis is a new type of programmed cell death which has been reported to be involved in the development of various cancers. In this study, we attempted to explore the possible links between ferroptosis and prostate cancer (PCa), and a novel ferroptosis-related gene prognostic index (FGPI) was constructed to predict biochemical recurrence (BCR) and radiation resistance for PCa patients undergoing radical radiotherapy (RRT). Moreover, the tumor immune microenvironment (TME) of PCa was analyzed.

**Methods:** We merged four GEO datasets by removing batch effects. All analyses were conducted with R version 3.6.3 and its suitable packages. Cytoscape 3.8.2 was used to establish a network of transcriptional factor and competing endogenous RNA.

**Results:** We established the FGPI based on ACSL3 and EPAS1. We observed that FGPI was an independent risk factor of BCR for PCa patients (HR: 3.03; 95% CI: 1.68–5.48), consistent with the result of internal validation (HR: 3.44; 95% CI: 1.68–7.05). Furthermore, FGPI showed high ability to identify radiation resistance (AUC: 0.963; 95% CI: 0.882–1.00). LncRNA PART1 was significantly associated with BCR and might modulate the mRNA expression of EPAS1 and ACSL3 through interactions with 60 miRNAs. Gene set enrichment analysis indicated that FGPI was enriched in epithelial–mesenchymal transition, allograft rejection, TGF beta signaling pathway, and ECM receptor interaction. Immune checkpoint and m6A analyses showed that PD-L2, CD96, and METTL14 were differentially expressed between BCR and no BCR groups, among which CD96 was significantly associated with BCR-free survival (HR: 1.79; 95% CI: 1.06–3.03). We observed that cancer-related fibroblasts (CAFs), macrophages, stromal score, immune score, estimate score, and tumor purity were differentially expressed between BCR and no BCR groups and closely related to BCR-free survival (HRs were 2.17, 1.79, 2.20, 1.93, 1.92, and 0.52 for cancer-related fibroblasts, macrophages, stromal score, immune score, estimate score, and tumor purity, respectively). Moreover, cancer-related fibroblasts (coefficient: 0.20), stromal score (coefficient: 0.14), immune score (coefficient: 0.14), estimate score (coefficient: 0.15), and tumor purity (coefficient: −0.15) were significantly related to FGPI, among which higher positive correlation between cancer-related fibroblasts and FGPI was observed.

**Conclusion:** We found that FGPI based on ACSL3 and EPAS1 might be used to predict BCR and radiation resistance for PCa patients. CD96 and PD-L2 might be a possible target for drug action. Besides, we highlighted the importance of immune evasion in the process of BCR.

## Introduction

Prostate cancer (PCa) has been the most common non-skin malignant tumor among American men since 1984, accounting for 26%, and the second leading cause of cancer deaths (Siegel et al., 2021). Radical treatment is one of the most common treatments for localized PCa, which mainly includes radical prostatectomy (RP) and radical radiotherapy (RRT). However, despite the improvement of techniques and perfect selection of indications for patients, 27–53% of men still encounter biochemical recurrence (BCR) (Amling et al., 2000; Hull et al., 2002; Roehl et al., 2004; Van den Broeck et al., 2019). Moreover, although thought to be a slowly growing tumor, about 10–70% of PCa patients experience recurrence after receiving RRT, indicating that some subsets of PCa cells possess radiation resistance (Allen et al., 2007; Weber et al., 2009). For recurrence patients, the median time to metastasis is 8 years, and the median time from metastasis to death is 5 years (Pound et al., 1999). In the most recent update, the median time for metastasis-free survival was 10 years (Antonarakis et al., 2012). Once a recurrence occurs, management of the tumor often becomes tricky. Thus, early prediction helps the realization of individualized precision medicine. At present, Gleason score is the most important prognostic factor that affects the biological behavior of tumors and predicts patient response to treatment (Epstein et al., 2016). D'Amico et al. proposed in 2000 that the percentage of positive prostate biopsies added clinically significant information regarding time to failure of prostate-specific antigen (PSA) after RP along with Gleason score, preoperative PSA value, and staging together. However, there is still no good standard to individually predict radiotherapy resistance population and high-risk BCR population.

Ferroptosis is a new type of programmed cell death defined in 2012 as different from necrosis and apoptosis, which may be caused by metabolic stress, such as glutathione (GSH) depletion, and is characterized by the accumulation of iron-dependent lipid peroxides to a lethal level (Stockwell et al., 2017). The ferroptosis-related enzyme GSH peroxidase 4 (glutathione peroxidase, GPX4) is the only enzyme that can use GSH as an electron donor to reduce the toxic lipid hydroperoxide in the biofilm to the corresponding alcohol (Yang et al., 2014; Rohr-Udilova et al., 2018). While GSH reduces reactive oxygen species and reactive nitrogen species, which can induce the polyunsaturated fatty acids in the phospholipids of the biomembrane to produce a lipid peroxidation chain reaction, it causes significant peroxidative damage and cell death (Das, 2019). Therefore, both the depletion of GSH and the decrease of GPX4 activity will cause the accumulation of lipid peroxides in the cells, thereby promoting the ferroptosis process. Overexpression of GPX4 in PCa cells inhibits cell cycle progression and cell migration by increasing GSH and reducing levels of reactive oxygen species, thereby inhibiting PCa progression (Rohr-Udilova et al., 2018). Simultaneously, GPX4 is the main target of ferroptosis inducers. Erastin can inhibit the activity of GPX4 by consuming glutathione, while RSL3 can directly inhibit the activity of GPX4 (Shimada et al., 2016; Jelinek et al., 2018). It was recently discovered that a second glutathione-independent protective pathway, FSP1/AIFM2, works in parallel with GPX4 during ferroptosis (Bersuker et al., 2019; Doll et al., 2019). At present, the research of antitumor drugs is mostly concentrated in the field of apoptosis, but almost all tumor cells will appear to have apoptosis resistance. But luckily, some highly malignant cancer cells have been shown to be innately susceptible to ferroptosis even after apoptosis resistance. All PCa cells are sensitive to ferroptosis-inducers, erastin and RSL3, even among castration-resistant PCa (Ghoochani et al., 2021). Therefore, induction of ferroptosis has been thought to be a new cancer treatment method (Zou et al., 2019).

It has been proved that ferroptosis occurs among various cancers, including liver cancer, lung cancer, pancreatic cancer, cervical cancer, osteosarcoma, head and neck carcinoma, and ovarian cancer (Lachaier et al., 2014; Sun et al., 2015; Hayano et al., 2016; Lin et al., 2016; Alvarez et al., 2017; Basuli et al., 2017). However, there are few studies linking ferroptosis to the development of PCa. Therefore, we tried to establish a ferroptosis-related gene prognostic index (FGPI) to predict BCR after RRT, and to predict radiation resistance and to limn the tumor microenvironment of PCa recurrence.

## Methods

### Data Collection

We merged GSE79021 (Sinnott et al., 2017), GSE32571 (Kuner et al., 2013), GSE62872 (Penney et al., 2015), and GSE116918 (Jain et al., 2018) from the Gene Expression Omnibus (GEO) database and removed batch effects (Edgar et al., 2002). We extracted the expression of messenger RNA (mRNA) and long noncoding RNA (lncRNA) from the combined datasets. The former three GEO datasets (Kuner et al., 2013; Penney et al., 2015; Sinnott et al., 2017) contained 248 normal and 476 tumor tissues which were used to identify the differentially expressed genes (DEGs) using the R package “limma” and the tumor-related genes by weighted gene co-expression network analysis (WGCNA). Tumor-related genes were defined as the absolute value of coefficient ≥0.3 and p.adj. <0.0001, and DEGs were defined as the absolute value of logFC ≥0.4 and p.adj. <0.05. Prognosis analysis was conducted using 248 tumor patients undergoing RRT with complete BCR data in GSE116918 (Jain et al., 2018). Besides, we obtained the PCa data in The Cancer Genome Atlas (TCGA) from UCSC XENA (Goldman et al., 2020) and downloaded ferroptosis-related genes from the Genecards database (Stelzer et al., 2016). By taking the intersection of tumor-related genes, DEGs, and ferroptosis-related genes, we obtained the candidate genes. For PCa patients undergoing RRT in GSE116918 (Jain et al., 2018), ACSL3, EPAS1, and NEDD4L were identified through the Lasso regression analysis, and COX regression analysis was further performed using these three genes. Moreover, we observed that ACSL3 and EPAS1 were the independent gene factors of BCR through the COX multivariate analysis, and we subsequently constructed the FGPI based on the coefficients of the two genes. The FGPI = −0.930*ACSL3-1.902*EPAS1. In addition, we enrolled the FGPI and clinical features in GSE116918 (Jain et al., 2018) into the COX regression analysis and further confirmed that the FGPI was an independent prognostic factor for BCR. 70% of patients from GSE116918 (Jain et al., 2018) were randomly extracted to internally verify the FGPI’s prognostic value of BCR. In addition, 425 PCa patients undergoing RP with complete prognostic data of metastasis in TCGA database were used to externally validate the prognostic value of the FGPI score. GSE53902 (Li et al., 2020) sequenced nine radio-resistant and nine normal DU145 cell lines, which were used to externally confirm the FGPI’s prediction value of radiation resistance. Moreover, we analyzed the relationship between immune indicators and FGPI score.

### Gene Interaction Analysis and Function Enrichment Analysis

We explored the protein–protein interaction of ACSL3 and EPAS1 through the GeneMANIA database (Warde-Farley et al., 2010). We identified lncRNAs which were differentially expressed between 248 normal and 476 tumor tissues in GSE79021 (Sinnott et al., 2017), GSE32571 (Kuner et al., 2013), and GSE62872 (Penney et al., 2015) and associated with BCR in GSE116918 (Jain et al., 2018). Subsequently, we established the interaction network of competing endogenous RNA (ceRNA) and transcription factor through lncBase (Paraskevopoulou et al., 2016), miWalk (Sticht et al., 2018), and TRRUST (Han et al., 2018). We analyzed the potential biological functions and signaling pathways through Gene Ontology (GO) and Kyoto Encyclopedia of Genes and Genome (KEGG) analysis of candidate genes. GO analysis consisted of biological process, cell composition, and molecular function. We divided the PCa patients undergoing RRT from GSE116918 (Jain et al., 2018) into high- and low-risk groups according to the median of the FGPI score. Gene set enrichment analysis (GSEA) of high- and low-risk groups from GSE116918 (Jain et al., 2018) was conducted as well. “c2.cp.kegg.v7.4.symbols.gmt” and “h.all.v7.4.symbols.gmt” were downloaded from the molecular signature database to evaluate related pathways and molecular mechanisms (Liberzon et al., 2011). P. adj. <0.05 and false discovery rate <0.25 were considered statistically significant.

### Drug Analysis and TME Analysis

**FIGURE 1:**
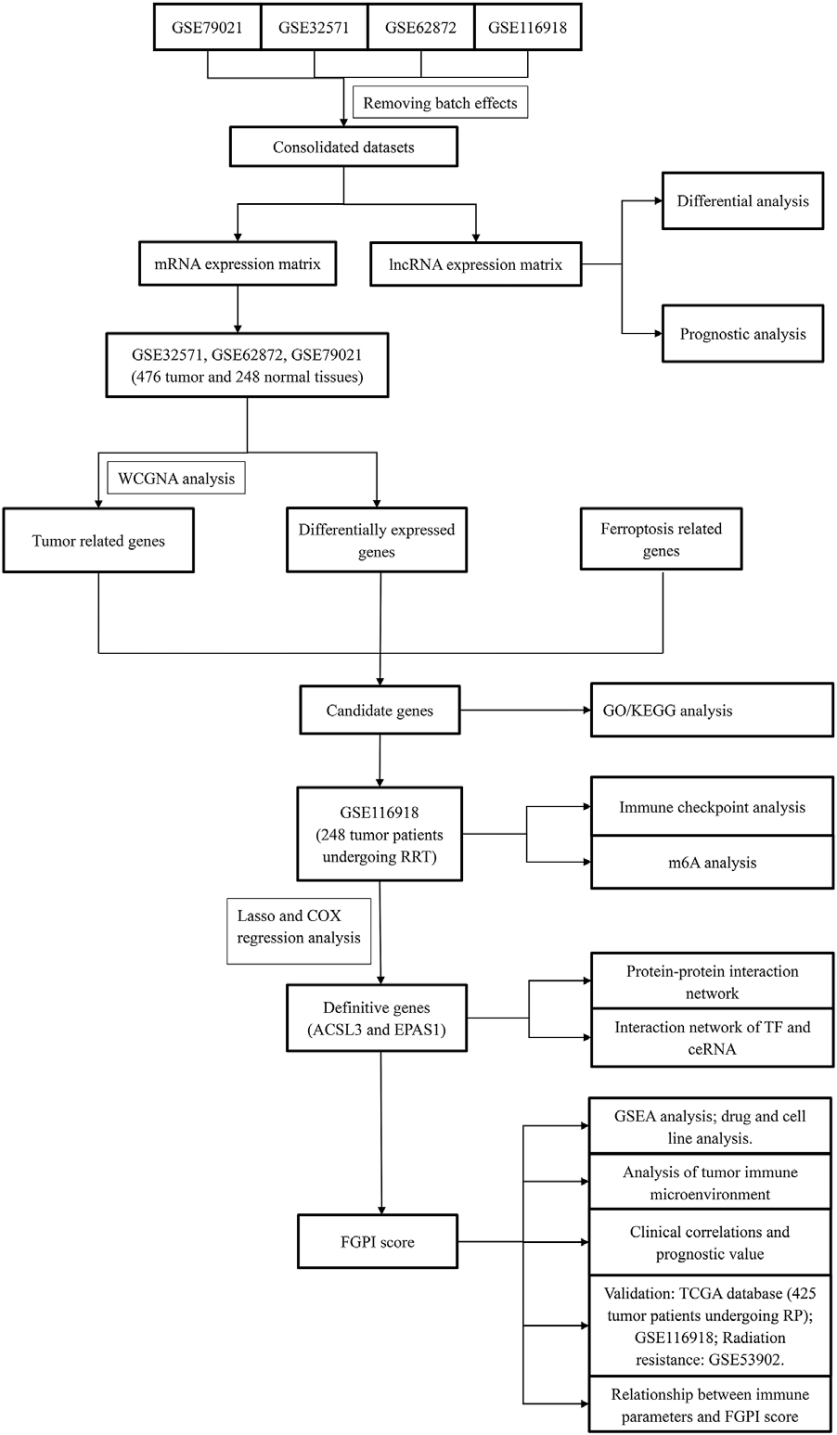
Flowchart of this study. WGCNA = weighted gene coexpression network analysis; GO = Gene Ontology; KEGG = Kyoto Encyclopedia of Genes and Genome; GSEA = gene set enrichment analysis; FGPI = ferroptosis-related gene prognostic index; mRNA = messenger RNA; lncRNA = long noncoding RNA; RP = radical prostatectomy; RRT = radical radiotherapy.

We analyzed the drug sensitivity of ACSL3 and EPAS1 through GSCALite which conducted drug analysis from the Cancer Therapeutics Response Portal (CTRP) and genomics of drug sensitivity in cancer (GDSC) (Liu et al., 2018). We analyzed the differential expression of the 17 common immune checkpoints in BCR and no BCR groups. EPIC (Racle et al., 2017), ESTIMATE (Yoshihara et al., 2013), and immunophenoscore (IPS) (Charoentong et al., 2017) algorithms were used to assess the TME of GSE116918 (Jain et al., 2018) through the R package “IOBR” (Zeng et al., 2021). Similarly, differential expression of TME-related cells in BCR and no BCR groups and their relationship with FGPI score were analyzed as well. We presented the flowchart of this study in Figure 1.

### Statistical Analysis

All analyses were conducted with R version 3.6.3 and its suitable packages. Cytoscape 3.8.2 was used to establish ceRNA and transcription factor network. BCR-free survival was the primary outcome, and metastasis-free survival (MFS) was the secondary outcome. The Wilcoxon test was used if data distribution does not satisfy normality. Only variables that are statistically significant in the univariable Cox regression analysis were included in the multivariable Cox regression models. Each outcome was regarded as statistically significant with two-sided *p*-value <0.05. Significant mark: ns, *p* ≥ 0.05; *, *p* < 0.05; **, *p* < 0.01; ***, and *p* < 0.001.

## Results

### Data Presentation and Clinical Value

**FIGURE 2:**
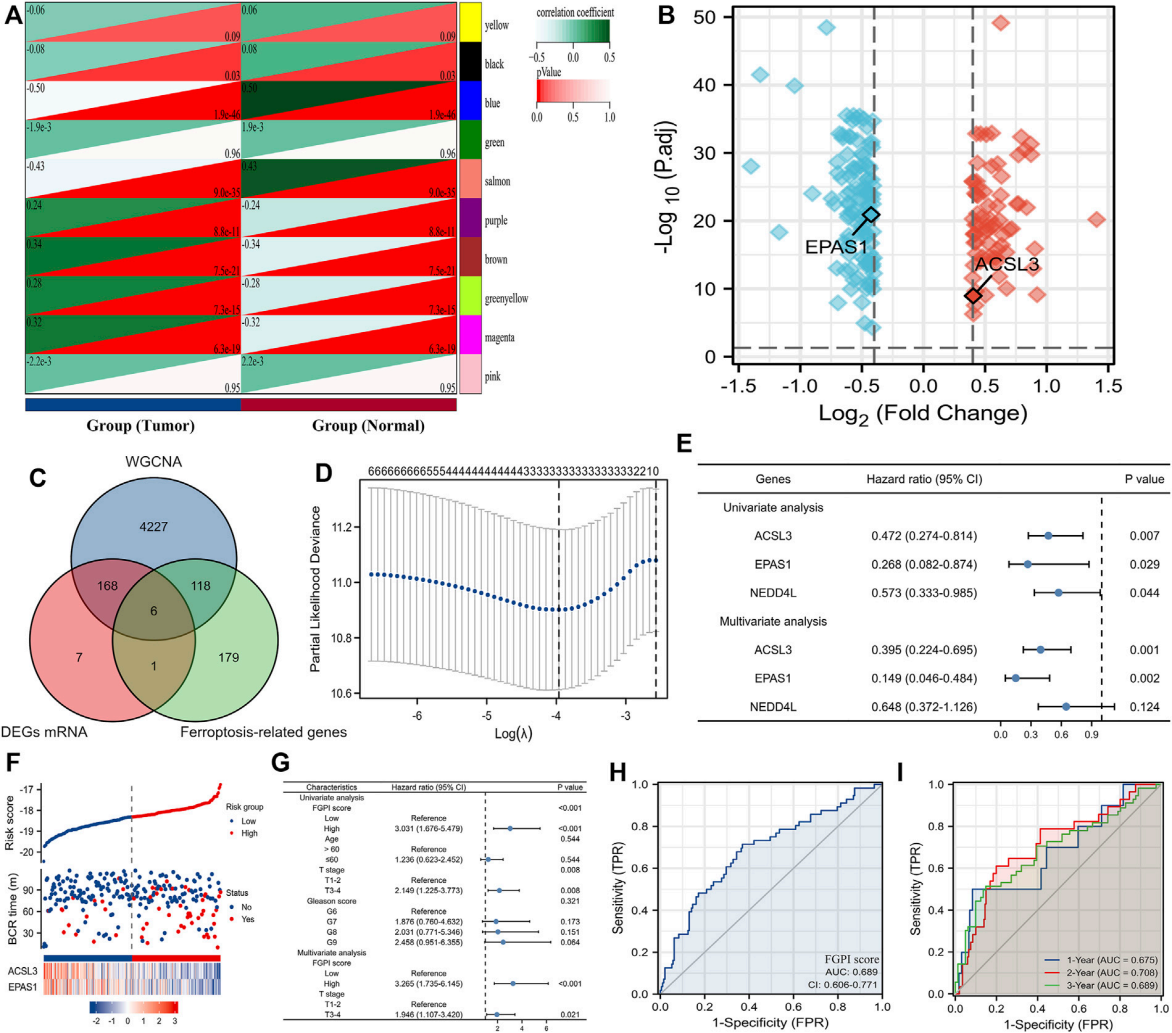
Identification of the FGPI score. **(A)** Modules and phenotype showing tumor-related genes using blue, salmon, brown, and magenta modules; **(B)** volcano plot presenting the results of DEGs between 248 normal and 476 tumor tissues in the three GEO datasets (Kuner et al., 2013; Penney et al., 2015; Sinnott et al., 2017); **(C)** Venn diagram showing the results of the intersection of tumor-related genes, DEGs, and ferroptosis-related genes; **(D)** identifying three genes (ACSL3, EPAS1, and NEDD4L) using the Lasso regression analysis; **(E)** identifying genes independently associated with BCR in GSE116918 (Jain et al., 2018) using the COX regression; **(F)** risk factor plot showing the prognostic data and mRNA expression of ACSL3 and EPAS1; **(G)** determining the prognostic value of the FGPI score for BCR-free survival through the univariate and multivariate COX analyses including the clinical features of PCa patients undergoing RRT in GSE116918 (Jain et al., 2018); **(H)** ROC curve discriminating BCR from no BCR for PCa patients undergoing RRT in GSE116918 (Jain et al., 2018) using the FGPI score; **(I)** time-dependent ROC curve discriminating BCR from no BCR for PCa patients undergoing RRT in GSE116918 (Jain et al., 2018) using the FGPI score. FGPI = ferroptosis-related gene prognostic index; ROC = receiver operating characteristic; BCR = biochemical recurrence; GEO = Gene Expression Omnibus.

We merged the four GEO datasets by removing batch effects (Supplementary Figure S1) (Kuner et al., 2013; Penney et al., 2015; Sinnott et al., 2017; Jain et al., 2018). We used the WGCNA analysis to identify the tumor-related genes through the three GEO datasets (Kuner et al., 2013; Penney et al., 2015; Sinnott et al., 2017). The blue, salmon, brown, and magenta modules contained 4519 genes associated with the tumor according to the abovementioned definition (Figure 2A). We used the volcano plot to present the results of DEGs between 248 normal and 476 tumor tissues in the three GEO datasets (Kuner et al., 2013; Penney et al., 2015; Sinnott et al., 2017). ACSL3, EPAS1, FASN, GSTP1, LDHB, and NEDD4L were identified as the candidate genes through the intersection of tumor-related genes, DEGs, and ferroptosis-related genes (Figure 2C). We enrolled ACSL3, EPAS1, and NEDD4L identified from the Lasso regression analysis (Figure 2D) into the COX regression analysis to identify the genes which were independent risk factors of BCR for patients undergoing RRT in GSE116918 (Jain et al., 2018) (Figure 2E). The risk factor diagram shows the difference in the distribution of the two gene expressions among the high- and low-risk groups (Figure 2F). We observed that FGPI score was an independent risk factor of PCa patients through COX multivariate analysis (HR: 3.265, 95% CI: 1.735–6.145; Figure 2G). The ROC curve showed that our FGPI score had low diagnostic accuracy (AUC: 0.689; Figure 2H) in discriminating BCR from no BCR within 1, 2, and 3 years (Figure 2I).

**FIGURE 3:**
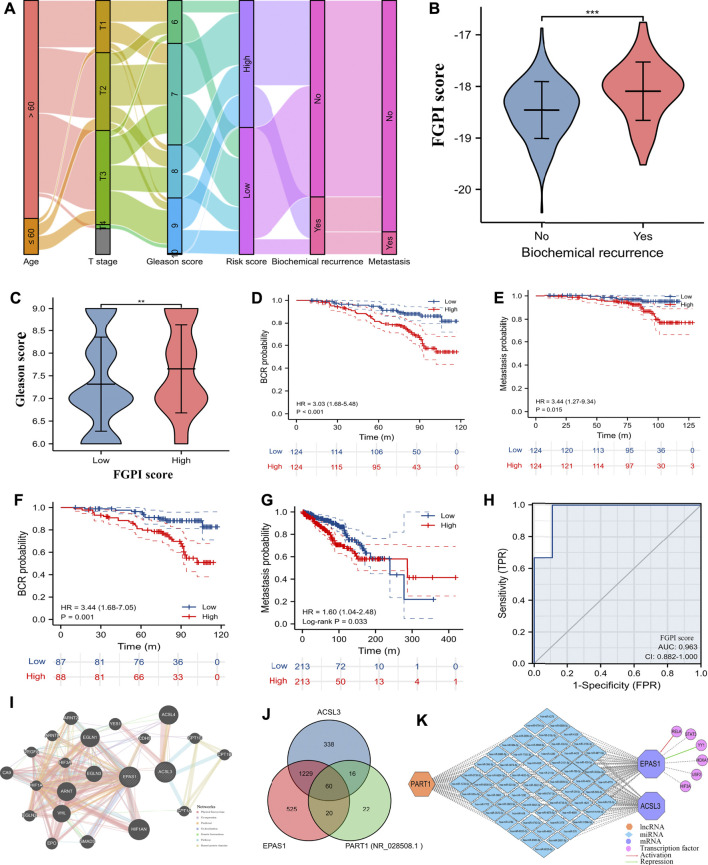
Clinical values and interaction network. **(A)** Sankey plot showing flow trend of clinical data and outcomes in GSE116918 (Jain et al., 2018); **(B)** comparison of FGPI score between BCR and no BCR groups for PCa patients in GSE116918 (Jain et al., 2018); **(C)** PCa patients in GSE116918 (Jain et al., 2018) were divided into high- and low-risk groups based on the median of the FGPI score and comparison of Gleason score between high- and low-risk groups; **(D)** Kaplan–Meier curve showing the difference of BCR-free survival for PCa patients undergoing RRT in GSE116918 (Jain et al., 2018) according to the median of the FGPI score; **(E)** Kaplan–Meier curve showing the difference of metastasis-free survival for PCa patients undergoing RRT in GSE116918 (Jain et al., 2018) according to the median of the FGPI score; **(F)** Kaplan–Meier curve of the internal validation showing the difference of BCR-free survival for 70% of PCa patients undergoing RRT in the GSE116918 (Jain et al., 2018) according to the median of the FGPI score; **(G)** Kaplan–Meier curve showing the difference of metastasis-free survival for PCa patients undergoing RP in TCGA database according to the median of the FGPI score; **(H)** ROC curve showing the diagnostic accuracy of the FGPI score for radioresistance; **(I)** protein–protein interaction network showing genes might interact with ACSL3 and EPAS1 using the GeneMANIA database (Warde-Farley et al., 2010); **(J)** Venn plot showing potentially interacting miRNAs of ACSL3, EPAS1, and PART1; **(K)** interaction network of competing endogenous RNAs and transcription factors. ROC = receiver operating characteristic; FGPI = ferroptosis-related gene prognostic index; BCR = biochemical recurrence; RRT = radical radiotherapy; RP = radical prostatectomy.

The flow trend of clinical data and outcomes in GSE116918 (Jain et al., 2018) was presented. (Figure 3A). We observed weak correlation between FGPI score and age (r: 0.130, *p* = 0.048). For PCa patients undergoing RRT in GSE116918 (Jain et al., 2018), BCR patients had a significantly higher FGPI score than no BCR patients (Figure 3B), and patients in the high-risk group had a significantly higher Gleason score than those in the low-risk group (Figure 3C). The Kaplan–Meier curve showed that higher FGPI score was an independent risk factor for BCR (HR: 3.03, 95% CI: 1.68–5.48; *p* < 0.001; Figure 3D) and MFS (HR: 3.44, 95% CI: 1.27–9.34, *p* = 0.015; Figure 3E) for PCa patients undergoing RRT in GSE116918 (Jain et al., 2018). In internal certification using the patients from GSE116918 (Jain et al., 2018), we observed a similar result (HR: 3.44; 95% CI: 1.68–7.05; *p* = 0.001; Figure 3F). For PCa patients undergoing RP in TCGA database, we detected that patients in the high-risk group were at higher risk of metastasis than those in the low-risk group (HR: 1.60, 95% CI: 1.04–2.48; Figure 3G). The ROC curve showed a strong diagnostic value of FGPI for the radiation resistance of PCa (AUC: 0.963, 95% CI: 0.882–1.000; Figure 3H). Through the GeneMANIA database (Warde-Farley et al., 2010), we found that ACSL3 and EPAS1 might work together through coexpression and other genes (Figure 3I). In the ceRNA network, the lnRNA PART1 might modulate the expression of ACSL3 and EPAS1 through 60 miRNAs (Figures 3J,K). RELA could activate the expression of EPAS1, while YY1 could suppress the expression of EPAS1 (Figure 3K).

### Function Enrichment Analysis

**FIGURE 4:**
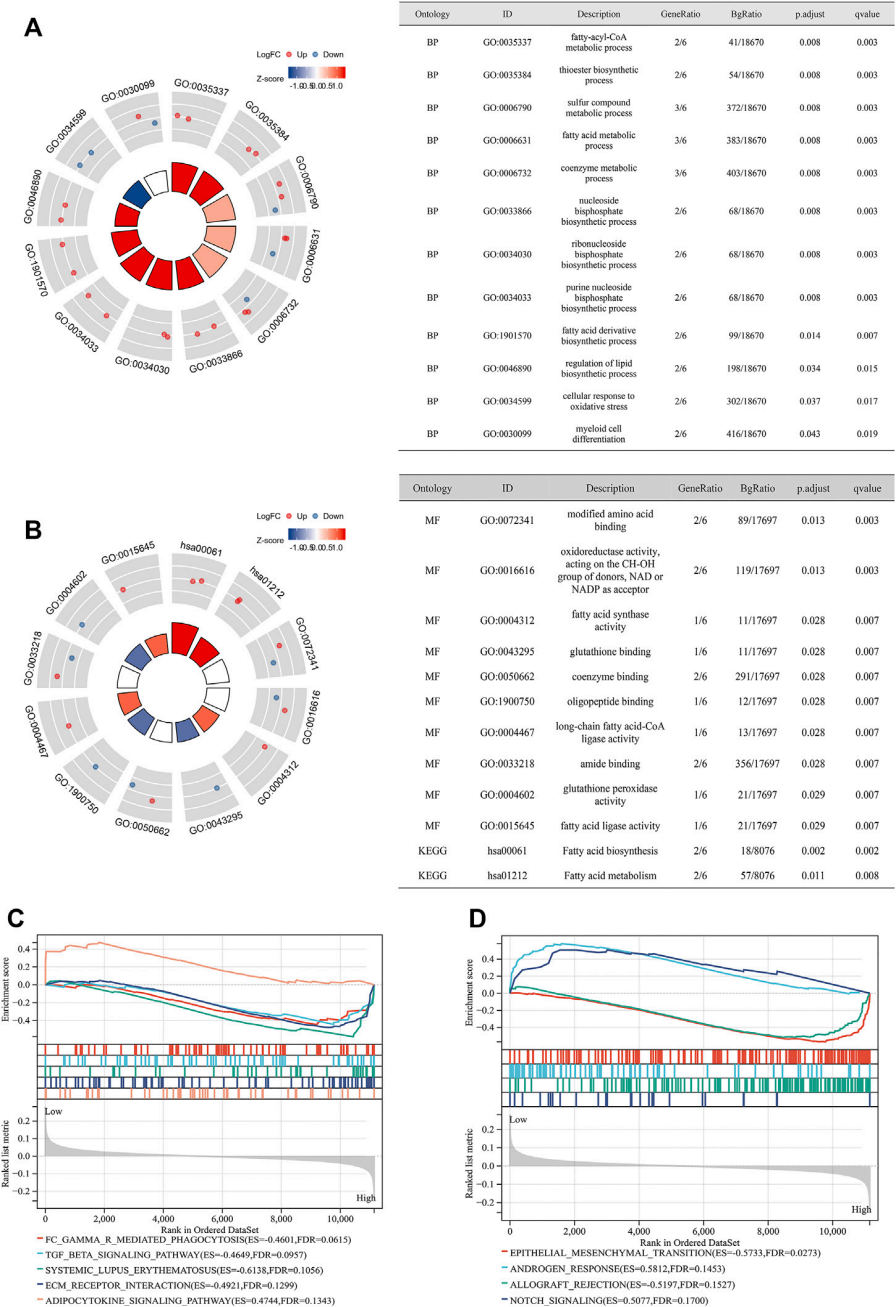
Functional enrichment analysis. **(A)** GO analysis showing the results of BP; **(B)** GO analysis showing the results of MF and KEGG analysis; **(C)** GSEA analysis of high- and low-risk groups (according to the median of the FPGI score) for PCa patients undergoing RRT in GSE116918 (Jain et al., 2018) using the subset of “c2.cp.kegg.v7.4.symbols.gmt;” **(D)** GSEA analysis of high- and low-risk groups (according to the median of the FPGI score) for PCa patients undergoing RRT in GSE116918 (Jain et al., 2018) using the subset of “h.all.v7.4.symbols.gmt.” GO = Gene Ontology; KEGG = Kyoto Encyclopedia of Genes and Genome; GSEA = gene set enrichment analysis; BP = biological process; MF = molecular function.

GO and KEGG analyses indicated that the candidate genes are mainly engaged in the fatty-acyl-CoA metabolic process, nucleoside and ribonucleoside bisphosphate biosynthetic process, regulation of lipid biosynthetic process, cellular response to oxidative stress, and fatty acid biosynthesis and metabolism, as well as GSH binding, coenzyme binding, and fatty acid ligase activity (Figures 4A,B). GSEA analysis showed that high-risk patients were enriched in epithelial–mesenchymal transition, allograft rejection, Fc gamma R-mediated phagocytosis, TGF beta signaling pathway, and ECM receptor interaction, while low-risk patients were enriched in adipocytokine signaling pathway androgen response and notch signaling (Figures 4C,D).

### Drug and Immunologic Analysis

**FIGURE 5:**
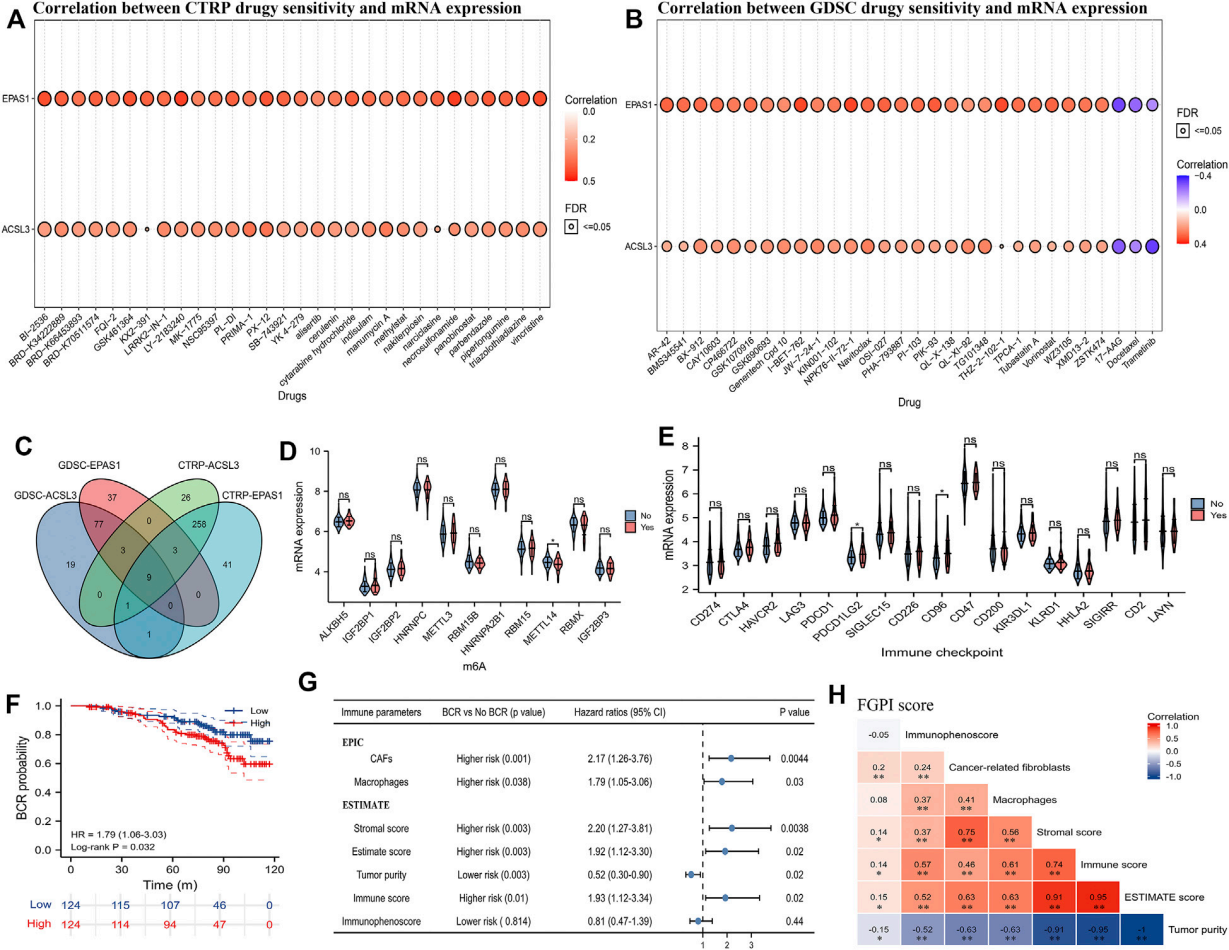
Drug sensitivity analysis and TME analysis. **(A)** Plot showing the top 30 potentially sensitive drugs to ACSL3 and EPAS1 using the CTRP; **(B)** plot showing the top 30 potentially sensitive drugs to ACSL3 and EPAS1 using the GDSC; **(C)** Venn plot showing the commonly sensitive drugs to ACSL3 and EPAS1 in the CTRP and GDSC; **(D)** violin plot showing the difference of the mRNA expression of m6A genes between BCR and no BCR groups for PCa patients in GSE116918 (Jain et al., 2018); **(E)** violin plot showing the difference of the mRNA expression of immune checkpoints between BCR and no BCR groups for PCa patients in GSE116918 (Jain et al., 2018); **(F)** patients in GSE116918 (Jain et al., 2018) were divided into high- and low-expression groups according to the median score of CD96, and Kaplan–Meier curve presented the difference of BCR-free survival for high- and low-expression groups; **(G)** forest plot showing the difference between BCR and no BCR groups and prognostic values of the TME parameters for PCa patients in GSE116918 (Jain et al., 2018); **(H)** heatmap showing correlation among FGPI score and TME parameters. GDSC = genomics of drug sensitivity in cancer; CTRP = the cancer therapeutics response portal; TME = tumor immune microenvironment; FGPI = ferroptosis -related gene prognostic index; CAF = cancer-associated fibroblasts; BCR = biochemical recurrence.

We presented the top 30 correlations between CTRP drug sensitivity and mRNA expression of ACSL3 and EPAS1 in pan cancer (Figure 5A), as well as the correlation between GDSC drug sensitivity and mRNA expression of ACSL3 and EPAS1 in pan cancer (Figure 5B). We identified nine potentially sensitive drugs through intersection of GDSC-EPAS1, GDSC-ACSL3, CTRP-ACSL31, and CTRP-EPAS1 (Figure 5C). The nine possible drugs were OSI-027, OSI-930, PAC-1, PHA-793887, PI-103, PIK-93, SNX-2112, TPCA-1, and UNC0638. m6A analysis showed that METTL14 was expressed significantly lower in the BCR group than the no BCR group (Figure 5D). Immune checkpoint analysis showed that mRNA expression of PDCD1LG2 (PD-L2) and CD96 was significantly higher in the BCR group than that of no BCR groups (Figures 5D,E). We divided the 248 PCa patients from GSE116918 (Jain et al., 2018) into high- and low-risk groups according to the median of the expression of PD-L2 or CD96. Through the survival analysis curve, we found that only CD96 was significantly associated with BCR-free survival (HR: 1.79; 95% CI: 1.06–3.03; Figure 5F). For TME analysis, we observed that between BCR and no BCR groups, cancer-related fibroblasts, macrophages, stromal score, immune score, estimate score, and tumor purity were differentially expressed and were risk factors for BCR (HRs were 2.17, 1.79, 2.20, 1.93, 1.92, and 0.52 for cancer-related fibroblasts, macrophages, stromal score, immune score, estimate score, and tumor purity, respectively; Figure 5G). Moreover, cancer-related fibroblasts (coefficient: 0.20), stromal score (coefficient: 0.14), immune score (coefficient: 0.14), estimate score (coefficient: 0.15), and tumor purity (coefficient: −0.15) were significantly related to FGPI (Figure 5H).

## Discussion

Compared with other cancers, PCa exhibits very special metabolic characteristics, such as early reliance on mitochondrial metabolism and dysregulation of both fatty acid synthesis and oxidation pathways, which may endow PCa cells with the characteristics of ferroptosis tendency. The 10-year overall survival and cancer specific survival of RRT was comparable to RP in prospective trials (Wallis et al., 2016; Wallis et al., 2018). And, salvage radiotherapy (SRT) is the first choice after BCR (Boorjian et al., 2009; Wiegel et al., 2009). The main challenge of PCa management is not the lack of initial treatment options, but treatment adaptation leading to resistance. It is generally agreed that the goal of defining BCR is to determine an early sign of treatment failure before clinical treatment. In the era of precision medicine, it is a new method to predict BCR based on individual tumor-related genes for selecting RRT indications. However, there is a lack of effective prediction and management measures for the problems of RRT resistance and BCR.

Ferroptosis is an attractive and very promising concept that can combat the resistance of various types of cancer to multiple therapies. Ferroptosis is considered a natural barrier to the progression of various cancers (Xu et al., 2019; Bebber et al., 2020). Recent research has focused more on the application of ferroptosis inducers (such as erastin) to treat drug-resistant castration-resistant prostate cancer (CRPC) (Yi et al., 2020; Ghoochani et al., 2021). However, there are few studies linking ferroptosis to BCR and radiation resistance among PCa. Previous studies have found that the overexpression of Tip60, LDH5, AKR1C3, miR-191, etc. confers PCa resistance to radiotherapy (Koukourakis et al., 2014; Sun et al., 2016; Xie et al., 2018). In this article, we first determined an FGPI comprising ferroptosis-related genes, ACSL3 and EPAS1, which has valuable and independent significance for predicting both BCR and radiation resistance. ACSL3 has been found to contribute to the growth of PCa in the mouse model, and high expression of ACSL3 is associated with poor prognosis (Obinata et al., 2016). Furthermore, some researchers have discovered that ACSL3 can mediate the activation of monounsaturated fatty acids and promote the function of oleic acid, thereby potently inhibiting ferroptosis of tumor cells (Magtanong et al., 2019; Ubellacker et al., 2020). Interestingly, in ovarian cancer, however, the homozygous deletion of ACSL3 is significantly related to the increased risk of recurrence in patients treated with adjuvant chemotherapy, suggesting that ACSL3 may be cancer-specific and possess complicate functions (Jeong et al., 2017). EPAS1 is a hypoxia-inducible gene encoding hypoxia-inducible factor 2α (HIF-2α). Research by Peng et al. showed that EPAS1 siRNA nanoparticles can inhibit the proliferation of pancreatic cancer cells and induce apoptosis under hypoxic conditions (Peng et al., 2017). EPAS1/HIF2α may be a key transcriptional control target for tumor cells to respond to hypoxia, which is a characteristic of aggressive tumor cells, and is also involved in resistance to chemotherapy and ionizing radiation (Peng et al., 2017; Påhlman and Mohlin, 2018).

In this study, we observed that the lncRNA PART1 mediated the expression of EPAS1 and ACSL3 and was significantly associated with BCR in PCa. Multiple studies have found that the overexpression of the lncRNA PART1 promoted the infiltration of mononuclear immune cells, prediction to current immune checkpoint gene markers, inhibition of tumor proliferation, promotion of cell apoptosis, and suppression of cell invasion in bladder cancer (Hu et al., 2019). At the same time, the lncRNA PART1 is also involved in the progression of renal clear cell carcinoma (Liu et al., 2019; Zhou et al., 2021). The epithelial–mesenchymal transition (EMT) plays an important role in the progression of PCa, characterized by morphological changes in their phenotype from cuboidal to spindle-shaped (Montanari et al., 2017; Odero-Marah et al., 2018). Research by Yan et al. showed that in the EMT model of PCa, circRNAs regulate EMT through PI3K-Akt signaling and TGF beta signaling pathways (Yan et al., 2020). Meanwhile, the oncogenic activation of the PI3K-AKT-mTOR signaling pathway has been shown to be related to the inhibition of ferroptosis *via* the sterol regulatory element-binding protein-mediated lipogenesis (Yi et al., 2020). Now, we speculated that based on our results, the interaction of the lncRNAs PART1, EPAS1, and ACSL3 may promote EMT through PI3K-Akt signaling and TGF-β signaling pathways, thereby causing the occurrence of BCR in PCa, with ferroptosis as one of the important links in this process. The complicated mechanism requires further study.

CD96, which is mainly expressed in natural killer (NK) cells and T cells, acts as a vital checkpoint in immunity and tumor progression, with CD96 mice displaying hypersensitive NK-cell responses to immune challenge and significant tumor resistance (Blake et al., 2016). By blocking CD96 in mice, the growth of primary tumors was inhibited, thus promoting greater tumor control (Mittal et al., 2019). CD96 has already been clearly associated with the prognosis of human hepatocellular carcinoma, advanced ovarian cancer, and glioma (Sun et al., 2019; Liu et al., 2020; Maas et al., 2020; Zhang et al., 2020). In pan-cancer analysis, CD96 was found to affect immune cell infiltration and malignant properties, thereby significantly affecting the prognosis of various cancers (Ye et al., 2021). This antitumor immune function of CD96 may be derived from the interaction of CD155/CD112 on tumor cells or antigen-presenting cells in the TME with TIGIT/CD96 on CD8 T cells or NK cells, resulting in the inhibition of antitumor NK cells and T cell functions (Dougall et al., 2017). The study by Du et al. showed that in PCa, CD96 can affect the late phase of immune response and PCa development through physical interaction with the PCa risk locus 8q24 (Du et al., 2015). In our research, CD96 was first found to be related to the time when BCR occurred in PCa. We speculate that CD96 may weaken the antitumor immune function of NK cells and CD8 T cells in the TME, achieve the purpose of immune evasion, make BCR occur early, and seriously affect the prognosis of patients, which makes CD96 a potential curative and preventive target for BCR. Another immune checkpoint, PD-L2, is closely related to immune-related pathways and radiation response pathway, and also possesses the ability to predict the response to postoperative radiation therapy (Zhao et al., 2019). Combined with our research, we speculate that PDL2 is also one of the immune checkpoints and indicators for evaluating prognosis and resistance for PCa.

The TME of PCa is a complex cancer-promoting and suppressing environment, which includes cancer cells and non-cancer cells, such as fibroblasts, immune cells, endothelial cells, and normal epithelial cells (Joyce and Pollard, 2009). This mixture is believed to play an important role in tumor progression and resistance (Hanahan and Weinberg, 2011; Junttila and de Sauvage, 2013). PCa is generally defined as “cold tumor,” which means the immune cells in the TME are lesser (Danaher et al., 2018). Our research shows that the higher immune score, the lower the purity of the tumor, the greater the probability of BCR, and the lower the BCR-free survival. This may partly be attributed to the immune evasion mechanism mentioned above. Meanwhile, the inflammatory microenvironment, characterized by a high immune score and low tumor purity, will increase the mutation rate, and with the recruited white blood cells and lymphocytes expressing cytokines and inflammatory factors that promote tumor progression and induce angiogenesis and hypoxia, a tumor-friendly microenvironment is established (Soucek et al., 2007; Mantovani et al., 2008; Grivennikov et al., 2010; Aran et al., 2015). For example, the tumor-associated macrophages (TAMs) in the TME are the main source of cytokine secretion, among which M1 macrophages express high levels of pro-inflammatory factors TNF-α, IL-1, IL-6, IL-12, or IL -23, along with the M2 phenotype, as the main body of TAMs, expressing anti-inflammatory cytokine IL-10, scavenger receptor A, and arginase, thus promoting tumor angiogenesis, invasion, metastasis, and tissue remodeling (Condeelis and Pollard, 2006; Mantovani et al., 2008; Sica et al., 2008). Moreover, in cancer stroma, CAFs, as the main component, can induce a cancerous phenotype and support tumor epithelial growth, invasion, and therapeutic resistance (Tripathi et al., 2012). The previous study showed that high density of CAFs might be associated with advanced-stage disease, higher Gleason scores, lymphatic metastases, higher PSA, and poor BCR-free survival in PCa patients (Wu et al., 2021). Besides, prostatic CAFs could induce tumorigenesis in normal human prostatic epithelial cells *in vitro via* the secretion of CXCL12, and this mechanism was found to be dependent on the presence of TGF-β *in vivo* in a mouse model (Fiard et al., 2021). Consistent with the previous studies, in this study, we found that CAFs were highly associated with the poor BCR-free survival of PCa patients and TGF-β was also highly enriched in the high-risk group. Combined with our research results, it is reasonable to believe that non-tumor cells in the TME, especially macrophages and fibroblasts, serve as seeds (cancer cells) in the soil (TME) and interact through stromal–epithelial interactions, which can promote the colonization of tumor cells and occurrence of PCa resistance (Hart and Fidler, 1980; Paget, 1989). The development of treatments that target TAMs and CAFs to prevent the growth of seeds *in situ* and its colonization at secondary sites is the focus of treatment goals. At the same time, the immune-related, gene-based FGPI we developed can well reflect the characteristics of the immune microenvironment and predict BCR and MFS.

The previous study explored the role of ferroptosis-related genes in predicting BCR for PCa patients undergoing RP in TCGA database (Lv et al., 2021). They enrolled nine ferroptosis-related genes (AIFM2, AKR1C1, AKR1C2, CBS, FANCD2, FTH1, G6PD, NFS1, and SLC1A5) into the model. In this study, we identified two different genes (ACSL3 and EPAS1) to predict the BCR-free survival for PCa patients undergoing RRT. We thought that the different sequencing methods, treatments, and data proceedings contributed to different conclusions. For the first time, we identified genes related to BCR and radiation resistance of iron death in PCa patients undergoing RRT and established a predicted FGPI. Our results suggest that ACSL3 and EPAS1 genes and related pathways are a method of iron death parallel to apoptosis, which is a potential therapeutic target for the treatment of drug-resistant PCa, and also an indicator for predicting the occurrence of BCR. CD96 and PD-L2 are the two immune checkpoints of PCa, and drugs targeting these two cell surface molecules may be effective in preventing BCR. Finally, the tumor microenvironment of PCa is also one of the research focuses of BCR in PCa. Despite these promising results, we have the following limitations. First, gene expression signatures are subject to sampling bias caused by intratumor genetic heterogeneity. Second, the microenvironment features might be distinct in different tumor regions, such as tumor core and invasive margin. More importantly, all findings, such as the ceRNA network and radioresistance in this study, still warranted to be further confirmed.

## Conclusion

We found that FGPI based on ACSL3 and EPAS1 might be used to predict BCR and radiation resistance for PCa patients. CD96 and PD-L2 might be a possible target for drug action. Besides, we highlighted the importance of immune evasion in the process of BCR.

## Data Availability

The original contributions presented in the study are included in the article/Supplementary Material, further inquiries can be directed to the corresponding author.

## Abbreviations

APCs, antigen-presenting cells; BCR, biochemical recurrence; CAF, cancer-associated fibroblast; ceRNA, competing endogenous RNA; CRPC, castration-resistant prostate cancer; GEO, Gene Expression Omnibus; GPX4, glutathione peroxidase; GSH, glutathione; lncRNA, long noncoding RNA; mRNA, messenger RNA; NK cells, natural killer cells; PCa, prostate cancer; RP, radical prostatectomy; RRT, radical radiotherapy; SRT, salvage radiotherapy; TAMs, tumor-associated macrophages; TCGA, The Cancer Genome Atlas; TME, tumor immune microenvironment.
