# Supplementary material for: A Ferroptosis-Related Gene Prognostic Index Associated With Biochemical Recurrence and Radiation Resistance for Patients With Prostate Cancer Undergoing Radical Radiotherapy
Source: Front Cell Dev Biol. 2022 Feb 10;10:803766. doi: 10.3389/fcell.2022.803766 (PMC8867172; doi:10.3389/fcell.2022.803766)
Supplement: Supplementary file 1 [file Table1.docx]

Supplementary figure 1. Nomogram plot for patients undergoing radical radiotherapy in the GSE116918 [30].


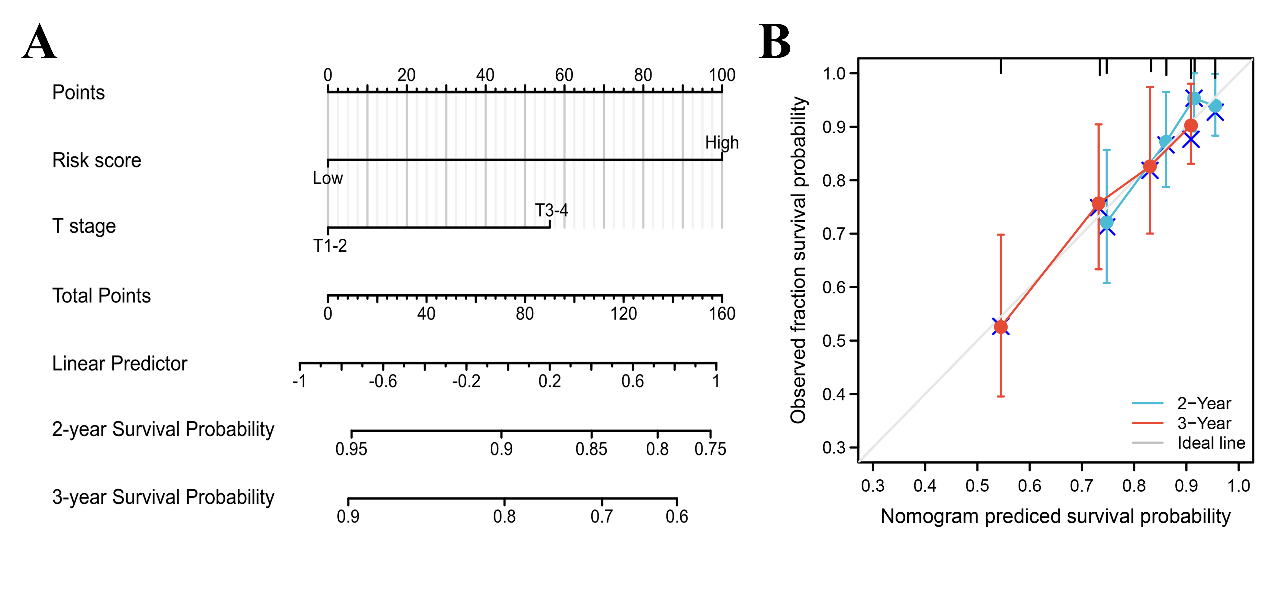

(A) nomogram plot for biochemical recurrence-free survival; (B) calibration plot
